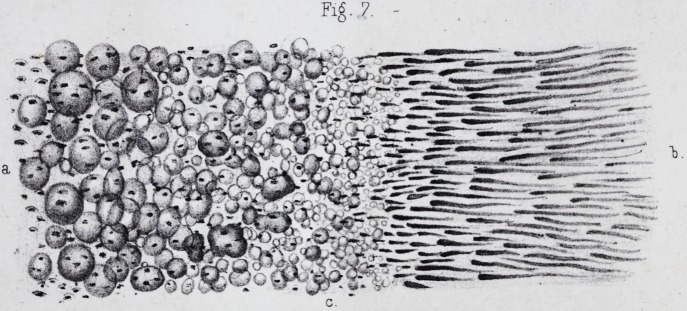# Magitot on the Development and Structure of the Human Teeth

**Published:** 1859-10

**Authors:** 


### AMERICAN JOURNAL

OF

### DENTAL SCIENCE.

Vol. IX. NEW SERIES—OCTOBER, 1859. No. 4.

### ORIGINAL COMMUNICATIONS.

### ARTICLE I.

*Magitot on the Development and Structure of the Human Teeth.* Translated for the American Journal of Dental Science.

(Continued from No. 3, July, 1859.)

THE whole mass of dentine thus created undergoes still further molecular changes, by which dentine globules, with varied and multiplied, anastomoses result, producing the canaliculi. But these subjects have been discussed above.* †.

* See last paragraph of this translation in the July No.—*Tr.*

† We believe that general anatomy may throw much light on this subject, and we will, therefore, enter into some minute considerations :

Organized matter, (formed or unformed anatomical elements) besides the properties which it possesses in common with unorganized matter, enjoys special and exclusive properties. These fundamental properties or elementary acts are five in number :

1st. *Nutrition,* a vital, elementary property, produced by a double molecular movement of simultaneous assimilation and disassimilation. It is the condition of

Our theory then, approaches the opinion of Raschkow and Huxley, inasmuch as we consider dentine as belonging to a phenomenon of production to which even the existence of all the others, whilst the only conditions of its manifestation are the conditions, in a word, of a proper, physical or chemical menstruum.

2d. *Development,* a vital, elementary property, which consists in this, that all organized substances, which are properly nourished, grow and terminate their existence in death.

3d. *Genesis,* generation or production. The fundamental property which determines the generation or production of analogous or like substances in the vicinity of organization. Or in other words, all organized matter possesses the property of birth—the property of appearing when some instants before it did not exist.

4th. *Contractility.*

5th. *Innervation.* (Ch. Robin, *Anat. Generale.*)

The three first fundamental properties, called vegetative, because they are found united in vegetables, are the only ones of the anatomical class of products which become perfect, (see the preceding note,) and it is remarkable that the phenomena of nutrition, development and genesis, are endowed in products with an activity which we do not meet with in constituents.

We know, in short, with what energy and rapidity the evolution of the epithelium is effected, and we may observe in certain species of animals, the rodents, for example, that the extraction of a tooth is soon followed by the generation of new formative elements which reproduce the primitive organ. *Products* also possess another property of importance: great resistance to putrefaction. This advantage is not enjoyed by constituents.

Thus, on the one hand, we have vital, vegetative, active properties; on the other, an unchangeableness almost absolute; such are the characteristics of products. As to the constituents, besides the three first properties, they sometimes also possess the two last, which are called animal properties.

All the anatomical elements, without exception, possess the vegetative properties, but only a small number are endowed also with the two animal properties, and none of them is simultaneously the seat of the two last. Never, indeed, are the five vital properties observed in the same species: *contractility* and *innervation* are constantly inherent to distinct species of elements, and are never found united in one.

Thus all organized substances, by the conditions of existence, possess the properties of nutrition, development and genesis. These fundamental acts, however, require for their accomplishment certain physical or chemical conditions foreign to vital movement. Conditions of another order are sometimes necessary to the formation of certain elements. Thus the production of the ovule requires, normally at least, the presence of an ovary; that of bone, the presence of a cartilage of ossification; that of dentine, the presence of a special organ, the dental pulp. But the ovule element, the osseous element, the dental element, is created within or at the surface of the organ which precedes it, and which represents, only in another way, the essential condition of their formation.

Bone is not produced by simple calcareous incrustations of the cartilage of ossification, but by the osseous elements developing within the elements of the cartilage, by which the absorption and final disappearance of this last is effected.

tissue of the pulp remains completely foreign, if it is not as the source of blastema; but it is different in many important respects:—1st, in the fact, that we deny entirely

As for the dentine, the phenomenon is different, and the cells are produced at the surface of the pulp, which furnishes, from its wall, the blastema necessary to their formation without participating directly in any other way.

Let us now examine, if the tooth, placed in the class of products, possesses the three vital properties. If we can prove their presence, we shall establish that the tooth is a living body, for the union of these properties constitutes life without the intervention of the other two.

We see the dentine cells, in fact, created at the surface of the dental pulp within a blastema exuded by this organ. This birth or generation is manifest. Once produced we observe a nutritive movement, which is proven by the modification and density which occurs in the soft cell. It becomes a mass of dentine of great hardness. Its resistance increases with the progress of age by continual assimilation of calcareous materials, carried by the canaliculi and their divisions; this assimilation, the first part of the complex process of nutrition, ends in disassimilation, which seems very limited, and which would appear to be incompatible with the functions of resistance to which the teeth are called; but this disassimilation does exist; experiments lately made by M. Flourens, seem to have established it, and we observe it, moreover, in great intensity under the form of total or partial reabsorption, which the roots of the provisory teeth undergo physiologically; pathologically, the same thing occurs with the permanent teeth.

The dentine cells, the formative elements of the tooth, then, are *born*, are *nourished*, since they present the double movement of composition and decomposition which characterise this phenomenon—and are *developed*, since by their evolution they become ivory.

But this development is not complete, as we have said, if the elements, which are the seat of it, are not liable to a death, end or termination. Let us establish then, the notion of the death of the anatomical elements in general, and determine if it is applicable to the dental elements.

The anatomical element, once created, once produced, it might be supposed to present a perfect equilibrium of infinite duration, between the act of assimilation and that of disassimilation. It might also be supposed to abruptly discontinue these acts, by which its existence would be speedily terminated. One may obtain this end by placing this element in certain conditions which arrest or render impossible, the double act of which we speak. The principal phenomena which provoke this result are, liquefaction, softening, and atrophy. But there is another way of terminating the elements, which though, consisting in a cessation of nutrition or molecular organic renovation, is attached in a much more intimate manner, than the preceding to its development. This mode is peculiar to *products;* it consists in this fact, that certain anatomical elements enjoy an activity of assimilation which the slowness or arrest of disassimilation fails to counterbalance. The death of the organ is the result, by the preponderance of material over other immediate principles of organized substance: this preponderance, arriving at this point, causes the disappearance of the conditions of immediate composition requisite to its nutrition, that is to say, to the continuity between assimilation and disassimilation; examples: nails, hair, epithelium, etc." (Ch. Robin, *Traité d'Anat. génér.*, inedited communication.)

The ivory elements end in this last manner. The assimilation of the calcareous the existence of the *membrana preformativa*, or any distinct membrane of the bulb, primitively situated on the surface of this organ and playing any part in the development of the tooth ; 2d, because, instead of the fibres of Raschkow, and the calcareous molecules of Huxley, we substitute certain cells, the characteristics of which we have described. But the main point of the doctrine is with Huxley as with us—to wit: the non-participation of the histological elements of the pulp. This theory has received from Huxley the name of *theory of deposition.* The elements of the tooth being deposited, according to this theory, between the pulp and the *membrana preformativa;* but this term does not seem to us to exactly represent the essential phenomenon of the production of the dental organ, the spontaneous generation of the elements which compose it, and we would prefer to adopt the term of *doctrine of autogenesis*, which, signifying the birth or genesis of the tooth, would precisely describe this physiological act. We propose this term without reserve, and leave it to our judges and the public to decide its scientific value.

Be that as it may, if we survey the different theories which have succeeded in science, we perceive that the human mind has followed in this case the same inevitable logic which becomes necessary to conduct the creation and progress of general anatomy. Before the seventeenth century, indeed, anatomists were governed by false analogies, and looked upon the teeth as bone.

At a later period, when nails, hair, horns and hoofs, were admitted to be the products of secretion, and when the presence of secreting membranes were supposed to be materials, which begins with the cells, continues within the organ until advanced age, and as an inverse phenomenon does not respond sufficiently to this incessant assimilation, it follows that the tooth acquires very great hardness; the canaliculi diminish in diameter, and are even obliterated in their finest ramifications; the central cavity, in its turn, disappears under the absorption of the pulp and the production of ivory which fills it, and then the death of the organ follows.

every where, the dental pulp was thought to be a glandular organ, the tooth a product of secretion, and even in France, we know, this doctrine, more or less modified, met with the greatest success. In short, a science in which Leeuwenhoeck, in 1683, had already been successful, again appeared, and soon justified its pretentions to the determination of the different terms of the problem which occupies us; microscopical anatomy unites then, in a single group, modern anatomists who demand of it the secret of the production of the teeth. But, it will be said, if general anatomy gives the complete solution of the problem, how will the numerous dissidences between micrographers be explained, and who will be induced to admit the different facts of different observers? Our reply is, that the dispositions observed are identical, the interpretation alone varies; that which Schwann calls cells, Raschkow considers fibres, from the cause, doubtless, that he saw them through weak magnifiers, and the subsequent disappearance of the nucleus. How is it that many authors consider the nucleus as a hollow cavity in the interior of the cell, when it is so easy to see that it is a solid, since one may sometimes meet it free, isolated, and broken into many fragments? (pl. i, fig. 7.) How is it that Kölliker and Lent state that the canaliculi are constituted by the prolongations of the dentine cells, whilst Tomes and Owen believe that they are formed by the juxtaposition of the cells and the union of their nuclei? These differences of opinion, it must be confessed, are very frequent, and we have, in the course of this work, sought to explain them; but should one accuse a science but yet in its infancy, or the precious instrument it employs? Assuredly not; the microscope, as M. Broca has well said, is as innocent of the errors of micrographers as the scalpel is of those of anatomists; and if this new voice does not conduct us to the whole truth, it should be confessed freely, whatever M. Oudet may say to the contrary, that from it alone are we at present to expect any part of it.

It is to this precious source that we owe our present convictions; it is, in a word, to the authority of observed facts, that we have left the care of deciding the intimate nature of the phenomenon which is the subject of our studies, and we are happy to say, in conclusion, that our master, M. Ch. Robin, has not ceased to protect our researches by his benevolent solicitude and his wise counsels, consecrating the results at which we have arrived and adopting the conclusions we have drawn.

### CHAPTER II.—*Development of the Enamel.*

### FACTS.

We have studied the disposition and structure of the enamel germ in another chapter, and as we already know that the entire face of this organ, which corresponds to the germ of the ivory, is covered by a range of cells (adamantine membrane of Raschkow and enamel membrane of F. Cuvier,) it will devolve upon us now to determine the characteristics and successive modifications by which these are transformed into fibres or prisms of enamel.

These cells thus united, have a prismatic form, which results from the pressure which they exert one upon the other, and which preserves this form in the columns of the enamel. When isolated they are usually cylindrical or of an elongated ovoidal shape. In length, from 0m 03 to 0m 05 ; in breadth they are extremely small, and vary from 0m 001 to 0m 003. They contain pale granulations and a constant nucleus, which occupies the centre of the cell. (pl. ii, fig. 2.) This nucleus is ovoidal, with a very clear and well defined contour, and a centre delicately granulated and brilliant, without very distinct nucleoli. Its volume relatively to the mass of the cell, is considerable, its transverse diameter sometimes reaching 0m 004, so that in an isolated cell, one may observe its edges passing the limits of the cell. We frequently meet with many free nuclei which warrant the supposition that like the ivory cell the nucleus pre-exists in the enamel cell, and that this latter is formed around it.

If we compare this description with the characteristics which we have assigned to the ivory cells, we will discover apparent resemblances. It is important, however, to distinguish clearly the two kinds of elements, and we shall, in this view, reconsider some points which serve to distinguish them. The enamel and dentine cells are all prismatic when they are united, but the first are longer and broader than the latter; the nucleus, which occupies the centre of the enamel cell, is nearly always situated at one of the extremities in the ivory cell, and the granulations which fill the latter are paler than those in the former. Finally, glycerin completely dissolves the ivory cell, but has no effect on the enamel cell beyond rendering it slightly paler.

The form and disposition of the cells of the enamel being thus determined, their transformation into enamel is very simple. Placed between the organ which has presided at their formation, the germ of the enamel of the first layer of dentine, the first cells arrange themselves upon the surface of the ivory and adhere there by one of their extremities, whilst the other remain adherant to the gelatinous mass of the organ of the enamel.* Thus perpendicularly arranged on the surface of the ivory, (pl. i, fig. 1, *E*,) the cells commence to undergo calcification: they then press one against the other, and their form becomes regularly prismatic. The nucleus gradually disappears under the invasion of calcareous matter into the cell which now presents the appearance of a six-sided prism with strongly pronounced edges. They are very transparent and fragile. At this period the enamel appears on the surface of the ivory as a layer of a chalky consistence,

* This extremity is believed by Nasmyth (*Researches*, p. 104,) and Hannover, (*loc. cit.*, p. 830,) to be provided with filiform prolongations. We have not been able as yet to satisfy ourselves of this.

composed exclusively of these imperfectly hardened prisms, which are frequently broken during the preparation, (pl. ii, fig. 3.) Subsequently, when the enamel is completely formed, the prisms are closely joined to each other without the interposition of any other substance, (pl. ii, fig. 5, *a*,) and when their calcification is thus complete, it is impossible to separate by the employment of acids.*

Such, in a few words, is the evolution of the enamel cells, and when the first layer has undergone its successive transformations, new layers are produced superimposed upon the preceding; they, in their turn, undergo the same modifications. The result is, that the enamel which covers the crown of the tooth, is composed of many concentric layers representing an equal number of cell rows.† (pl. ii, fig. 4, *a*.) In certain places, however, where the enamel is thin, the ivory is only covered by one layer of cells; we observe this at the necks of the teeth and at the anfractuosities of the triturant face of the molars. In these latter points, as in the crowns of eroded teeth, we still observe imperfections of structure which have been carefully studied by Tomes.‡

These imperfections are ordinarily occasioned by the accumulation of irregular, calcified, and granular prisms. The prisms arranged together in shapeless bundles, leave spaces between them, which becoming filled with air,

* The question as to the priority of the formation of the enamel and ivory, has been discussed by most writers; the greater number concur in the opinion that the ivory is formed first; Jourdain supports the contrary view. As for us, we have proven, in connection with M. Robin, that the ivory cells pass first from the dentine state, and that the cells of the enamel are created upon this last in proportion as dentification is effected. In a word, the cells of the enamel are produced, not by contact of the dental cells themselves, but on the surface of the newly formed ivory.

† According to Hannover, (*loc. cit.*, p. 833,) a single row of cells suffice to form the entire thickness of the enamel. This appears to us to be impossible, particularly as we can easily perceive, as we have seen in a vertical section of a tooth, (pl. ii, fig. 4, *a*,) concentric lines, in which we may count the number of layers which have been formed, each of which we have considered as representing a distinct layer of cells.

‡ See *Quarterly Journal* of *Microscopical Science*, Jan. 1856, p. 103, and *loc. cit.*, p. 54.

produce such fragility in the enamel, that this is soon broken by mastication, and leaves the ivory uncovered. Sometimes even the enamel may have undergone an arrest of development, and upon certain points of the crown be entirely wanting; furrows, fissures and holes result, which are the predisposing causes of dental caries.

When the evolution of the enamel cells is finished, and this tissue completely developed, the gelatinous part of the germ of the enamel progressively atrophies and disappears; but before this phenomenon is completed, a portion of the amorphous matter of the organ seems to condense upon the exterior surface of the enamel, under the form of a thin amorphous follicle, which we find later in the structure of this tissue, and which Kölliker has called the cuticle of the enamel. As to the pretended membrane which separates the ivory from the enamel, and which Cuvier believes he has discovered, no observation has yet demonstrated its existence. As we have seen from the preceding, the enamel obeys the same law of formation as the ivory; the creation and successive transformation of the cells differing in the two tissues, but undergoing corresponding phases of evolution.

### B. HISTORICAL.

Ancient authors are silent upon the mode of development of enamel. According to them the teeth are entirely a product of ossification, and the enamel an osseous layer, differing only in its greater density from the ivory. This explanation prevailed even to the time of the origin of the doctrine of secretion; but, from this moment, the development of the enamel was submitted to the influence of the doctrines which the development of ivory underwent. We have no intention of reviewing and discussing at length the various opinions which have arisen upon this subject. We shall limit ourselves to briefly exposing the different phases through which this theory has passed, from Rau to the present time.

Hérissant,* who appears to have been the first one in France to maintain the theory of Rau, believed that he had discovered in 1754, that the internal membrane of the follicle enclosed a considerable number of glandular vesicles, the office of which was to secrete a particular liquid, which, depositing itself upon the layers of formed ivory, hardened and constituted enamel. At a later period, G. Cuvier adopted Hérissant's views, and was soon forced to acknowledge the complete absence of the pretended glands of the internal membrane; nevertheless, he persisted in attributing to this the secretion of the enamel.

"When we open," says he,† "the capsule of a young tooth, we find small molecules of the future enamel still very slightly adhering to this capsule, and easily detached from it. . . . . . The enamel is then deposited upon the tunic of the dental pulp by the productions of the internal layer of the capsule, and it thus compresses it against the internal substance, called osseous, (the ivory,) which it separates from it, and soon this tunic becomes imperceptible in the hard part of the tooth, or at least it only appears upon the surface as a very fine gray line, which separates the enamel from the osseous substance."

It is reasonable to state after this description, that Cuvier recognized the existence of the adamantine, membrane, which F. Cuvier, his brother, has first described under the name of *enamel membrane;*‡ but instead of perceiving that it directly forms the enamel, he has attributed to it the part of simply receiving upon its surface the elements of this substance, and of afterwards atrophying. However that may be, Cuvier's theory has existed up to our time. Geoffroy Saint Hilaire, E. Rousseau, Bourdet,

* Recherchés sur la formation de l'émail des dents. (Memoires de L'Acad. Roy. des Sciences, 1754.)

† Ossements Fossiles, t. i, p. 5I2, 4th ed.

‡ Dents des Mammifères, p. 23.

Delabarre, MM. Duvernoy and Oudet, have adopted it; but MM. Serres and Dujardin incline towards the German opinion, and consider the enamel as a transformation of the enamel membrane, whose cells become filled with calcareous matter, and, they add, with Retzius and Nasmyth, that these cells are analogous to the epidermis.

In Germany, thanks to the early introduction of the microscope, the question of the development of the enamel has been attentively studied. Schwann,* like other authors, sought to define the nature of this phenomenon, and first determined clearly the structure of the adamantine membrane to be formed of special cells, which by calcification are transformed into prisms of enamel. Some dissidences arose however upon the exact nature of these cells. Müller and Raschkow considered them as fibres; the latter had already given this definition to the cells of ivory; Retzius, imitated by Nasmyth of England, regarded them as little membranous pouches. But the greatest number of authors adopted Schwann's opinion. Finally, more recently, Kölliker and Lent,† have put forth a new theory for the formation of enamel; according to these authors, this substance is formed under the enveloping membrane of the pulp, the organ of the enamel remaining completely foreign to this phenomenon, and then, by a rapid return to ancient ideas, they advance two hypothesis:

1st. The fibres of enamel result from a secretion of the adamantine membrane, a secretion which traverses the preformative membrane in the liquid state, afterwards becoming solid. (Lent.)

2d. The fibres of enamel are created from the ivory and result from a plasma exuded through the canaliculi. (Kölliker.)

Kölliker has comprehended the extreme weakness of these two explanations; for after having rejected the first, he discusses at length the probabilities of the second, and

* Mikroskopische Untersuchungen, tab. iii, fig. 4.

† Kölliker, loc. cit., p. 432.

finally concludes by confessing the impotency of science upon this point.

We would object to the first interpretation, that a plasma exuded through a membrane would not suffice to explain a distinct formation, and so regular a disposition of the columns of enamel, and we would reply to the second, that it necessarily gives to the enamel organ, the disposition and the nature of which are perfectly understood at the present time, a role of inaction and uselessness, which we could not admit. Besides, as Kölliker avows himself, the anatomical relations between the organ of the enamel and the young tooth, the identity of disposition and of dimensions between the cells of one and the prisms of the other; the progressive disappearance of the adamantine organ, coinciding with the development of the enamel, are signs which show the most intimate relations between these two parts. If to these considerations we add the results given by observation noticed before in this chapter, there will no longer be any doubt that the organ of the enamel is charged with the production of this tissue.

A Danish anatomist, Hanover, whose work has already obtained a certain notoriety in Europe, and whose opinions relative to the development of ivory we have examined, says,* that the germ of the enamel is only constituted by the adamantine membrane, whose cells calcify, and he considers the gelatinous part of this organ as the germ of the cement; adding that a particular membrane (*membrana intermedia*) separates these two parts. We reply to this theory that although an immediate contact exists between the enamel cells and the gelatinous mass, without any membranous interposition, it is very necessary to observe that because the cement occupies in man only the circumfernce of the roots of the tooth, we would not attribute its formation to an organ which only surrounds the crown, and which disappears at the moment when the development of the enamel is finished, when the cement commences to form.

* Loc. cit. p. 818.

In England, where the influence of Hunter's ideas is considerably diminished, the theory of the illustrious physiologist, which taught that the enamel is nothing more than the sediment of a liquid interposed between the tooth and the capsule, the capsulary membrane or other organ having no part in this phenomenon, has been rejected.* Professor Owen, in 1841,† very clearly determined the nature of the enamel organ, in which he distinguishes the adamantine membrane in the cells, and the *actinenchymatous* pulp, of which, he has studied the structure, and believes it unfurnished with vessels. This pulp or gelatinous part of the enamel organ, has been observed equally by Todd and Bowman.‡ These latter have even described and figured the disposition of the nuclei, from whence depart in rays, short, transparent fibres, and frequently anastomoses between them. They give to this tissue the name of *starred areolar tissue;* but while clearly establishing a very decided distinction between the cells of the adamantine membrane and the pulp which supports it, they do not explain the office of the latter, of which they have, however, very plainly proved the consecutive re-absorption to the formation of the enamel, and of which we know we can find fragments adherent to the capsule, where they have been taken by Goodsir for vascular vellosities of this latter.

More recently Huxley,§ claiming as well as Kölliker, the permanent existence of the preformative membrane, and stating that all the dental tissues are formed beneath it, refused to acknowledge in the development of the enamel, the intervention of the organ of the enamel, which he considers like the epithelium of the capsule, as *deposited* molecule to molecule, between the ivory and the preformative membrane which covers it.

* Histoire Naturelle des Dents, p. 66.
† Odontography, Introduction, p. 57.
‡ Physiological Anatomy, t. ii, p. 175.
§ On the Development of the Enamel. (Quar. Jour. of Med. Sci., April, 1856.)

Finally, Tomes, in a more recent work, has given a new solution of this problem. Attacking the weak points of Huxley and of Lent, he denies the intervention of the *preformative membrane* in the development of the enamel, which might be the result of the transformation of the cells of the adamantine membrane. He believes that the membranes isolated by the others from the surface óf the enamel in progress of development, (cuticle of the enamel,) under the influence of acids—are no other than the enamel itself, decalcified and membrani-form, and he strengthens this assertion by the following experiment.

Having prepared a thin section of enamel in a longitudinal direction, from the incisor of a rat by means of an excising file, and having submitted this preparation for some time to the action of hydrochloric acid diluted with water, (half,) he placed it under the microscope and then proved that the fragments of the membrane were raised from all points of the section, not only from the triturating surface of the enamel, but also from the faces resulting from the frictions caused in order to use the preparation; these membranes were perfectly clear and transparent, presenting in a word, all the characters assigned to *preformative membrane.* It appears from thence evident that it was the enamel itself, deprived of its calcareous salts, which formed the membranous shreds designated by authors; for we cannot admit, in this case, the presence of isolated membranes, even in the thickness of the enamel.

Such is the state of the science touching the manner of the development of the enamel, a problem less difficult but not less studied than the development of ivory. Upon this point no majority of authors appear to be governed by the same theories, or by any precise observation. In France the prestige of Cuvier's ideas have retarded the progress of our schools, and yet occupy us, while in Germany the supposed necessity of admitting the intervention of a special membrane in the development of the teeth, trammels the efforts students are making to resolve the different questions of odontogenesis.

As to our personal opinion, it has been set forth in the first part of this chapter. We will at this time simply confine ourselves to the remark, that the explanation of Tomes on the subject of *enamel cuticle* cannot be admitted; for, as will be seen in the description of the structure of the teeth, the *cuticle* really exists, but cannot be considered as decalcified enamel, as it can be seen under the action of acids to detach itself from the surface of the enamel long before this action gives to the tissue the membranous appearance. As to its origin, we have already given our views.

### Chapter III.—*Development of the Cement.*

The cement seems to have been noticed for the first time in the teeth of the calf by Leeuwenhoeck,* who has not however given any very precise description of it. In 1767, Tenon† observed it in the molars and the cavities of the incisors of the horse; he gave the first complete history of it and called it the *osseous cortical*, a name still employed until Cuvier's time. In 1780, Blake‡ repeated the observations of Tenon and described the cement under the name of *crusta petrosa*. Finally, Cuvier in 1803§ announced that the osseous cortical served to unite the different layers which composed the molar of an elephant and gave it the name of *cement*. But the first author who has noticed this substance in the teeth of man was Retzius¶ in 1836, although Frænkel had already suspected it the year before.

The opinions put forth by authors in regard to the development of the cement are two in number. The first, defended by Cuvier and all the French school, is that this substance is the product of a secretion of the follicle.

* *Continuatio epistolorum;* Lugduni Bataviorum, 1680.

† See *Memoires de l' Institute National,* Acad. des Sciences, t. i, 1796 to 1797.

‡ *De Dentium Formatione et Structura in Homine et bariis animalibus,* 1780. *Edinbourg.*

§ *Ossements Fossiles,* t. i, p. 520.

¶ *Bemerkungen über den eimern Bauder Zähne* (mull. Arch., 1837.)

The second, represented by all the microscopic school (J. Muller, Kölliker, Tomes, etc.) is that the cement is the result of the ossification of the external membrane of the follicle.

As far as the limited number of our observations and researches go, they do not permit us to establish positively our view ; nevertheless, we believe we should adopt one or other of the two opinions we have given ; we think, then, that the cement results from the transformation of a special organ subjacent to the follicular membrane. This organ, very distinctly observed by Hanover in the follicle of certain mammifers, does not appear, however, although this anatomist has supposed it to exist in man in the interior of the dental follicle, before the perfection of the crown, and we have seen in effect that the organ to which Hanover attributes the production of cement is the gelatinous part of the enamel germ.

We have not then been able to prove the presence of a *cement germ* around the crown, in the human follicle ; but we have clearly observed it in a fœtus of a horse, about two months after conception. Such are the results of this study which we have very briefly given here, in order to establish the non-participation of the follicular wall, reserving for special researches the profound study of this point of odontogenesis, a study which is rendered very difficult by the slight thickness of the cement and of the organ which forms it as well as the tardy period of its appearance. These circumstances explain the recent date of the discovery of cement in man (1836) and the uncertainty which exists upon this question.

In the horse, the dental follicle, examined at the moment of the commencement of dentification or a little before this period, is composed of five parts enclosed the one in the other without the interposition of any substance and moulded upon the organ which occupies the centre of it, the germ of ivory. These five parts are, in their order and appearance :

1st. The external envelop of the follicle.

2nd. The internal envelop of the same follicle.

3rd. The cement germ.

4th. The enamel germ, the structure of which presents the greatest analogy to the same organ in man.

5th. The ivory germ, equally analogous to that of the human follicle.

The cement germ in the horse, is situated under the envelop of the follicle, between the internal membrane of the latter and the germ of enamel ; it represents a sac without opening, moulded exactly upon the parts it covers, that is to say, upon the enamel germ, this latter being moulded upon the germ of ivory, which occupies the central point. This organ is from 1 to 2 millimetres in thickness, extremely transparent and gelatiniform ; its softness is extreme and its resistance is so feeble, that it is difficult to seize it with pincers.

In order to prepare it it is necessary to pass a blunt instrument under it and separate it from the subjacent parts ; placing a fragment of it then upon a plate of glass, we notice that in spite of its gelatinous consistence the needles could not lacerate it, which is not the case with the enamel germ. Examined with the microscope we perceive that its structure is laminous and presents some analogy with the web of the enamel organ in man ; the nuclei however are not so much pressed one upon the other, and the prolongations which proceed from it are more numerous, paler and more frequently anastomose. But the most remarkable peculiarity the structure of this organ offers, is the presence of numerous myéloplaxes, some of which are multi-cellular and very voluminous and others uni-cellulars. *

* M. Robin has given the name of myeloplaxes (from *μυελσς, marrow,* and *πλὰξ, plate,*) plates or layers of multiplied nuclei of the marrow of bone, to a particular anatomical element in the normal state, characterized by a very variable form and size (0m 020 to 0m 100) flattened or many sided, at first generally irregular or dentelated, pale, thin or thick and marked, composed of a finely spread gelatinous mass of ovoidal nuclei (from 2 to 20 or 30.) The nuclei are in length from 0mm, 009 and in breadth from 0m 005. In the normal state

The presence in this tissue of one of the accessory elements of marrow of bone offers a character of great importance, and permits us in advance to claim the ulterior osseous nature of the germ of cement.

This organ, perfectly applied to the enamel germ placed under it, follows it in all its circumference, in such a manner as to hide itself in the anfractuosities and furrows of the molars and incisors in order to cover them with cement; for we know that in the horse as in other pachydermata, the cement entirely covers the teeth, appearing at the period of eruption and worn out in the efforts of trituration, exposing the layer of enamel, which becomes worn also by the tongue, and finally discovers the ivory which forms the central part of the teeth, the same as in the follicle the germ of ivory forms the central organ.

The cement organ is provided with a very considerable number of vessels.

It will be perceived, after this description, that the cement in the pachydermata, is manifestly formed in the centre of a special organ, and the observations of Cuvier demonstrates the same peculiarity in the ruminants. It appears then reasonable to conclude that the cement of the teeth in man obeys the same law. Nevertheless, not having collected a sufficient number of facts upon this subject, we express ourselves with the greatest hesitancy, and do not put forth any theory; we limit ourselves to saying, that according to our observations, the envelop of the follicle appears completely foreign to the production of cement.

the myeloplaxes are found more abundantly in the marrow of the deploe and in spongy tissue than in the long bones; they are proportionally abundant in the marrow of the osseous points (of the fœtus) of new formation. We often find them adherent to the osseous substance of the canal or areoles filled with marrow, and moulding upon the irregularities of this substance. (Ch. Robin, *Dictionnaire de Nysten.*)

### PART THIRD.

### *Structure of the Teeth.*

In the histological study of adult teeth, we shall distinguish two parts; 1st, the study of the hard parts, dentine, enamel and cement; 2d, the study of the soft parts, the pulp or dental germ, and the alveolo-dental membrane. These two parts will each form the subject of a separate chapter.

### CHAPTER I.—*Hard Parts of the Teeth.*

#### SECTION I.—*Dentine, or Ivory.*

*Synonyms. Substantia eburnea, ebur;* the osseous substance of the ancients; ivory, (Hunter, Cuvier,) principal substance, (Duvernoy,) tubular substance, (J. Muller,) dentine, (R. Owen.)

The dentine or ivory, represents the most considerable part of the dental organ; upon this the two other tissues are moulded, the enamel covering the crown, the cement, covering the root. It follows, therefore, that it no where forms the exterior part of the tooth, if we admit the exception of the neck of the tooth; modern research, however, has shown that the cement is prolonged several millimetres upon the edge of the enamel, and indeed, several writers, (Owen, Nasmyth, Erdl and Henle,) believe it to extend over the whole surface of this substance, where it forms the membranous covering which Kölliker has described under the name of *enamel cuticle.*

In the descriptive point of view, the dentine is a yellowish, semi-transparent substance in the fresh tooth: of a sparkling whiteness, and possessing a nacreous lustre in the dry tooth. Its density is something between that of cement and that of enamel, and besides varies with age; as in the infant and in the adult it is relatively slight, while it becomes very considerable in old age, when the teeth are very hard and brittle.

The exterior face of the dentine is covered by enamel and dentine. In the crown it is covered with little hexagonal depressions, described by Owen and Huxley, which receive the extremities of the enamel prisms, and which give a remarkable reticulated aspect. The large number and regular arrangement of these depressions, permit us to compare the exterior surface of the ivory in the crown, to the appearance presented by the cornea of the eyes of certain insects. In the root, the external surface of the ivory is unequal and rough, covered by anfractuosities filled by the cement.

The internal face corresponds to the pulp cavity, and to the dental canals found in it; it is adjusted with great exactness to the parts which cover it and presents innumerable orifices of the canaliculi which open to the surface of the germ.

The dental cavity which contains the pulp presents, like the last, the same figure, and nearly the same volume, as the tooth on the exterior. This cavity, very large in infancy, when the pulp is voluminous, diminishes with age, a circumstance which may permit us to say that the growth in volume of the tooth continues; not, it is true, as with rodents, where this organ is called upon continually in mastication, and grows in length, being constantly forced out of the alveolus; this growth continues in man within the cavity of the pulp, the capacity of which becomes gradually weaker and weaker, finally disappearing entirely under the incessant productions of ivory.

The ivory is composed of a *fundamental substance*, pervaded by a large number of *canaliculi*.

The fundamental substance, formed by the calcified cells of the dentine, seems to be completely homogeneous and very delicately granulous when magnified; at no point does it preserve any trace of the primitive elements. However, if we may believe Retzius and Owen, one may find calcareous cells deposited in the intervals of the tubes in the ivory of some animals, the incisors of the horse, for example. Submitted to prolonged maceration in hydrochloric acid, the fundamental substance decomposes, first into large parallel fibres in the direction of the canaliculi, and then into smaller ones; this circumstance has caused a number of authors (Nasmyth, Raschkow, and M. Oudet) to believe that the tooth has a fibrous structure. But it is easy to perceive that these fibres are very irregular in form, and that they are purely artificial or accidental. They result, in short, from an arrangement of the tubes, which, directing them parallel to one another, intercept between them some portions of the dentine to which decalcification has given the appearance of fibres. The fundamental substance is distributed in all parts of the ivory in various proportions; thus, in the exterior parts subjacent to the enamel and cement, it is more abundant than in the neighborhood of the dental cavity where the canaliculi are extremely close together.

The whole mass of the ivory presents a stratified arrangement,* indicated in a vertical section taken from the exterior contour of the crown, by curved, parallel lines. These lines, called lines of contour by Owen†, (pl. ii, fig. 4, *b*,) and by Salter *contour markings*,‡ are generally very near together, and their interval measures the thickness of the layers of the primitive cells. We also observe, on a level with these lines, in the same section, the profile of

* The ancients had observed the stratified arrangement of the layers of dentine, and they proved it by submitting the teeth to incineration. They could also decompose them into a number of little concentric lamellas, which could be easily separated. They made the same experiments with bone, and, as they remarked that these two tissues, under the same influences, underwent the same divisions, they concluded their identity. At a later period, Cuvier (*Dictionn. des sc. med., Art. Dent.,*) Heusinger (*Histologie,* 1822, p. 201,) and J. H. Weber (Hilderbrant, *Anatomie,* t. i, p. 306,) definitely demonstrated the lamellar structure of the ivory.

† Odontography, p. 464, and plate 122, fig. 7, *l.*

‡ Quarterly Journal of Micro. Science, July, 1853.

the dentine globules and the interglobular spaces, (pl. ii, fig. 5, *d.*)

The contours of the globules and the spaces which they intercept, are not equally marked in the different teeth, for they become effaced to some extent by age, without, however, wholly disappearing; they may be seen in the teeth of old persons in the form of very fine, pale, curved lines.

The dentine is limited in exterior surface by a continuous layer of membranous black granulations, very varied in form, (pl. ii, fig. 6, *d*, and fig. 5, *l.*) This *granular layer*, (Tomes,) subjacent to the enamel and cement, has been taken for a mass of osseous corpuscles, in which the canaliculi terminate, by Retzius and J. Muller.* But an attentive examination demonstrates that although the granulations may be continuous with the terminal extremities of the canaliculi, they cannot be assimilated to the osseous corpuscles; they should rather be regarded as lacunæ within the thickness of the ivory, at its exterior limit, in order to favor the communications between the tubes which furrow this tissue. We also propose to call this granular layer the *anastomotic source* of the dental canaliculi which permit a free flow of the fluid of imbibition, and thus forming the organic movement. This proves that the aspect under which these cavities are presented is the same as that of the canaliculi. Thus, when these are filled by a liquid which renders them white and transparent, the little cavities also become white and transparent, and they become black and opaque when the tubes, filled with air, present the same color. Besides, it is easy in varying the focus of the microscope, to see the two parts freely communicating. This granular layer has been long known. According to Cuvier,† it represents the vestige of the membrane situated between the ivory and the enamel, and which he believed he had dis-

* See Manuel de Physiologie, t. i, p. 316.

† Ossements Fossilles, t. i, p. 33; 1821.

covered. More recently Hannover* has regarded it as the modified remains of the *membrana intermedia*, to which he has given the name of *stratum intermedium,* interposed between the cement and the ivory. But no anatomist has, up to the present time, attributed to it the rôle and nature which we have indicated.

The dental canaliculi, (calcified canals of Owen,) discovered in 1678 by Leeuwenhoeck, are microscopic tubes, hollowed in the thickness of the ivory, where they represent the intervals between the primitive cells,† (pl. ii, fig. 6, *f*; fig. 5, *e*.) They open into the dental cavity by an orifice immediately in contact with the surface of the pulp, and they direct themselves in rays towards the exterior surface of the tooth, presenting in their route, a great number of more or less pronounced sinuosities. Besides these partial sinuosities, we also observe general curves implicating a great number of tubes, which undergo two or three considerable inflections in their length. These sinuosities and inflections have been explained by M. Oudet‡ as the result of change undergone by the pulp during the development of the elements to which it gives birth.

The calibre of the tubes varies at different points. At their orifice, in the pulp cavity, it attains 0mm 005 ; but the least diameter is from 0mm 0015 to 0mm 002.§ In the terminal extremities of the canaliculi the calibre becomes so small that the least ramifications often escape observation. The extreme tenuity of these tubes might be urged

* Loc. cit., p. 914.

† We do not think we ought to stop our demonstration of the existence of the canaliculi here. They were described for the first time by Leeuwenhoeck. In 1835 the experiments of Muller and Retzius, showing the hygroscopic properties of ivory, permit us to see the tubular structure which the observations of Purkinje and Frœnkel afterwards demonstrated. If a doubt remains on this question, we would cite the beautiful injections of the canaliculi last made by Professor Gerlach, and of which we have had a fine specimen under our eye.

‡ *L' Union Medicale,* 2d of December, 1856.

§ The diameter of the tubes, studied by different writers, are as follows :— 0mm 004 (Henle,) 0mm 0023, (Retzius,) 0mm 0008 to 0mm 0015, (Linderer,) 0mm 0007, to 0mm 0023 (Krause,) 0mm 0013, in the neighborhood of the dental cavity.

as an argument against certain writers, who would compare them to the Haversian canals in osseous tissue. The smallest blood globules (0mm 006) are larger than these tubes.

The number of these tubes is so great that their walls are nearly in contact. From their origin, upon the surface of the pulp, they send forth lateral ramifications which ordinarily circumscribe the base of the cells, or masses of calcified dentine, which they represent, then they undergo a first bifurcation of branches which soon subdivide a great number of times, so that from one primitive trunk, according to Kölliker, they give rise to sixteen canaliculi.

These then proceed parallel to each other, without presenting, during a part of their course, new bifurcations; but when they have nearly reached the exterior surface of the ivory, they again divide into a number of ramifications which terminate in a grand anastomotic source, of which we have spoken above, (pl. ii, fig. 6, *d.*) The reciprocal anastomoses of the canaliculi are effected in different ways; ordinarily they are lateral or oblique, sometimes directly transverse, and finally some are transverse, (pl. ii, fig. 6, *e.*)

Have the dental canaliculi proper parietes?

J. Muller, in 1836, answered in the affirmative, founding his opinion on the greater transparency of the tube than the contour which limits it; Retzius has professed the same opinion; Kölliker, Tomes, Hannover and Duvernoy, are also in the same category. All of these writers believe that they have seen the membranous wall in the deep contour of the canaliculi; the last mentioned writer* says, that this wall is formed by the membrane which surrounds the pulp, and which continues in the canaliculi.

We cannot concur in this view, and agreeing with M. Dujardin, we believe the canaliculi to be without proper parietes. As to the deep contour of the tubes, we can

* Duvernoy, Dents de Musaraignes, p. 17.

offer another explanation. If, then, we examine the thinned edge of a young cap of dentine in process of evolution, and seen from its pulp facc, we may easily see that the large orifices of the canaliculi are all transparent; but if we observe a thin section of ivory prepared from an adult tooth, as it is nearly impossible to realize a section through the exact axis of a large number of canaliculi, it follows that the contour will be always more or less deep. It is then easy to remark that the tubes which are cut through, allow the light to pass freely through their opening and preserve a clear opening, whilst those which have been cut obliquely, have a contour more or less deep, and to us it has always appeared easy in ranging the focus of the microscope, to recognize that the shades which surround the orifices result from the obliquity relatively to the axis of the canaliculi. The mechanism of the formation of the tubes, besides, cannot explain the formation of the proper walls, since they result from spaces produced between the dental cells during the different phases of its existence.

The dental canaliculi are filled during life, with a transparent and colorless fluid, containing, according to Hannover, the calcareous material in dissolution. This is probably intended to affect the organic movement within the tooth; it is at least quite logical to believe, since no experience has given the demonstrations. Krukenberg* has stated, it is true, that this liquid undergoes a permanent movement through the substance of the ivory; but some writers have denied this circulation. We are very disposed, however, to believe that this liquid, charged with the materials of nutrition, bathes the canaliculi through their entire course, and effects the double act of assimilation and disassimilation; thus a certain exchange is established, sluggish it is true, but still favored by the arrangement of the canaliculi, and their multiplied anastomoses.

The teeth contain no trace of vessels or nerves; notwith-

* **Zur Lehre Vorn Röhrensysteme der Zähne und Knochen. Mull· Arch. 1849.**

standing the vascularity of the ivory having been advocated by some authors. M. Flourens, as we have seen, believes in the presence of nervous filaments. Retzius and Owen believe that they have found vessels in the tooth of the anarrhique* and the pike; but Oudet has remarked with reason, that these canals cannot be regarded as vessels, for they are filled by the divisions of the pulp, which permits that they may be considered as prolongations of the cavity which contains this last. As to the other cavities which have been met with sometimes in the thickness of the dentine, they seem to result from an arrest of the development or of imperfect calcification, and thus it becomes necessary to consider them as vices of conformation. That there are such cavities there can be no doubt, as they have been described by Czermak and Hannover, and of which we possess a very remarkable specimen in our collection; Tomes has compared them to the canals of Haver, and other writers have mistaken them for internal caries.

A particular sensibility has been, and is still, attributed to the ivory, notwithstanding the proven absence of nervous ramifications, this view is sustained on the ground that the teeth experience lively impressions of temperature, acids, &c., and perceive the physical qualities of bodies submitted to them, such as grains of sand, hair, etc.

This tactile sensibility is, however, entirely foreign to the ivory, and the phenomena alluded to should be attributed to the extreme facility with which this substance receives the slightest vibrations, which are carried to the pulp, a tissue extremely rich in nerves, which fills the central cavity: if the mechanism is still doubted, we would refer, with Cuvier, to the labyrinth (of the ear?) situated in the cranium of certain fishes, which receives and communicates the vibrations. It cannot be questioned that this is as delicate and sensitive as the teeth.

Chemically the dentine presents the following composition, according to Bibra:—

* Genus of Apodes.

ADULT INCISOR.

| | Man. |
|---|---|
| Organic substances, . . . . . | 28.70 |
| Inorganic substances, . . . . . | 71.30 |
| | 100.00 |

ADULT MOLAR.

| | Man. | Woman. |
|---|---|---|
| Phosphate of lime with traces of fluoride of calcium, . . . | 66.72 | 67.54 |
| Carbonate of lime, . . . | 3.36 | 7.97 |
| Phosphate of lime . . . . | 1.08 | 2.49 |
| Soluble salts, . . . . | 0.83 | 1.00 |
| Cartilage . . . . . | 27.61 | 20.42 |
| Fat, . . . . . . | 0.40 | 0.58 |
| | 100.00 | 100.00 |

### SECTION II.—*Enamel.*

The enamel, or vitreous substance, covers the external surface of the crown. It presents a variable thickness; at the triturating surface, and at the level of the tubercles it is thickest, gradually diminishing as it approaches the roots, terminating at the neck in a very thin edge, which ordinarily pushes under the upper portion of the cement, (pl. ii, fig. 4, *d*.)

The enamel is a milky white substance, varying in shade with different subjects; it is diaphanous and homogeneous in nature; chemically it is extremely rich in calcareous materials, giving it so great a degree of hardness, that it resists the file, and moreover, can be made to strike fire. (Nasmyth.) The intimate union of enamel with ivory, close arrangement of the elements which compose it, and its very great resistance to chemical action, constitute a powerful means of protection to the crown. Yet it is gradually removed under the effects of mastication, and

(sometimes) leaves the ivory of the crown uncovered. This circumstance has not the dangerous effects one would suppose, for it only (?) occurs in advanced age, when the dentine, having acquired a considerable degree of hardness, is able to resist the influences to which it is submitted.

The enamel which covers the crown is composed of a certain number of stratified layers in thickness from 0mm 05 to 0mm 10, each of which represents the thickness of a layer of primitive cells. The number of superimposed cells is variable, but at the tubercles there may be five or six; whilst at the level of the neck, only one layer often constitutes the whole thickness of the enamel. This substance may be regarded as composed of little caps, one fitted into the other; this stratification may be shown, in a vertical section of a tooth, by very pale lines analogous to those which furrow the ivory, and which may also be designated as lines of contour, (pl. ii, fig. 4, *a*, and fig. 5, *b*.)

The lower face of the enamel, covered by those rugosities which we spoke of in the section on dentine, comes in direct contact with dentine without the interposition of another substance; its external face, equally rough, is covered by a slight amorphous pellicle, first described by Nasmyth* under the name of *persistent capsule*, to which some writers have attributed a nature and office anterior to the development of the tooth. Raschkow, Bowman, Huxley and Kölliker, call this the *preformative membrane* of the pulp, whilst Hannover calls it the *membrana intermedia*. We have remarked above, that not having succeeded in isolating any membrane from the parts constituting the follicle, we are the more ready to believe that this *capsule* is formed posterior to the development of the pulp.

However, this pellicle, called the *enamel cuticle*, by Kölliker, is a thin membrane which cannot be separated from

* Medico-Chirurgical Transactions, Jan. 1839.

the surface of the enamel by means of acids.* It is transparent and slightly granular ; its least thickness is 0mm 001. If we may believe this last author,† it is not attackable by any of the acids, offering great resistance, and thus being an excellent protection to the tooth. If boiled in potassa or soda it swells slightly without separating, and the alkali used gives a partially soluble precipitate with hydrochloric acid in an excess of acid.

The enamel cuticle seems to be composed of azotised organic matter, impregnated with calcareous salts, for its combustion gives an ammoniacal odor, and leaves a residue of alkaline ash.

The enamel is composed of little columns preserving nearly the length of the primitive cells, or slightly surpassing them, (pl. ii, fig. 5, *a*;) they are about from 0mm 05 to 0mm 08, in length, and 0mm 002 in thickness. These columns, to which J. Muller‡ has wrongly given the form of needles pointed at both ends, are regular six sided prisms, whence it results that a transverse section of the united columns present the appearance of a mosaic composed of regular hexagonal pieces.§

The prism or columns of enamel are usually directed vertically to the surface of the dentine, upon which they rest. In those points where the surface is convex, the prisms lose their parallel arrangement, and diverge towards the exterior surface of the enamel. This circumstance

* This *cuticle*, which has not as yet been noticed by any writers in France, is very easy to isolate. For this purpose it is necessary to prepare a thin section from the crown of a tooth at its eruption or just before; in preparing, care should be taken to leave the free border of the enamel intact. Place it in a little water between two pieces of glass, and then use the microscope; add a drop or two of hydrochloric acid, and a thin membrane will soon be separated from the enamel layer.

† Histol. Hum., p. 422.

‡ Manuel de Physiologie, t. i, p. 317.

§ The structure of the enamel has been studied at an early period. Cayliardi (Anat. Oss.. p. 61, 1689) mentions a fibrous arrangement, an opinion which has been established in our day by Raschkow and several other writers. Malpighi (Opera Posthuma, p. 52, 1697) speaks of a filamentous arrangement; Hunter (Nat. Hist. of the Teeth) discovers a crystalline nature, and compares it to biliary calculus and vesicles.

gives place, towards the peripheral extremity of the prisms, to spaces, and little columns, which do not reach the ivory, occupy the spaces thus made. In the concave surface, on the contrary, the columns of the opposite sides of the concavity form angles by their union, or meet by their free extremities. In these two cases, the junction of the prisms is always imperfect, and this is proven by their meeting, by the changes of direction from whence irregular masses, arranged in different ways, result. These vices of conformation, which often occur in the concavities of the crown of the molars, sometimes form fissures at the surface of the enamel, at the bottom of which the ivory may be seen naked. These fissures, which we have already described in treating on the development of the enamel, were observed as early as 1699, by La Hire.* "In some teeth it happens," says he, "that the fillets which form the enamel united into packets, and which should touch at their extremities, are not exactly joined towards the interior part of the tooth; this is observable in the bone of molar teeth, where the separation of the packets may be seen. If the extremity of the fillets are rubbed a little, the separating of the two packets will increase so that hard portions of the aliment are received into the opening at the bottom of the tooth; the interior part of the tooth is exposed, and its death follows."

In their aggregate direction, the columns of the enamel have inflexions or parallel undulations, sometimes regular, resulting from general curves;—sometimes irregular, which gives a spiral or zigzag direction; these peculiarities are only observable in the thick part of the enamel, for in those where it is thin, the prisms are regularly parallel.

The junction of the enamel columns is often imperfect, so that we find numerous lacunæ in teeth badly conformed, in the region of the surface of the ivory. In this last

*** Memoires de l'Academie Royale des Sciences, 1699. See Fauchard, Art du Dentiste, t. i, p. 53.**

place they have the shape of elongated vacuities, and sometimes penetrate into the ivory, where some writers have stated that they receive the terminal canaliculi, of which they consider them as enlargements.

Other lacunæ are presented in the bundles of prisms, but we have never been able to see that cavity, which Tomes* has described, in the prisms themselves. These different vices of conformation have the grave inconvenience of predisposing the teeth to fractures and to traumatic lesion of the enamel.

The prisms of the enamel present transverse, reciprocal, parallel striæ, about from 0mm 002 to 0mm 004 apart. This arrangement, which some writers have compared to the striation of muscular fibre in animal life, has not seemed to us to be proven, and has been stated differently. According to Kölliker† and Tomes‡, these striæ are due to slight varicosities which the prisms undergo, longitudinally; Hannover‡ regards them as traces of isolated calcification of the cells: thus, says he, they are more apparent in young than in old subjects, in the teeth of whom more complete calcification has rendered them invisible. Duvernoy,‡ who has observed them in the teeth of musaraigns, finds them to give the prisms the appearance of oblong paving stones stuck together, and gives a similar explanation to that of Hannover, on their origin, at the same time stating that this arrangement is far from constant. As for us we have often observed these striæ in fibres of enamel in process of development, or in the adult tissue treated by hydrochloric acid, which separates the elements; we are not able, however, at present, to pronounce upon the mechanism of their formation.

The enamel once developed, undergoes a very slow organic movement. But this movement exists; we are not indeed ignorant of the changes of density and coloration

* A Course of Lectures on Dent. Surg., p. 53.

† Histol. hum., p. 420.

‡ Loc. cit., p. 56.

|| Loc. cit., p. 28.

to which this tissue is liable during the course of life, nor of the fragility the tooth acquires when removed from the economy and consequently dead.

The experiments of Rutherford and Flourens have established, it is true, that the enamel is never colored by madder, but if we consider, as it has been studied by M. Robin, that the anatomical elements which compose the shells and calcareous envelops of molluscs, present an internal organic movement of composition and decomposition, we cannot be far wrong in admitting as much of enamel. This tissue seems besides, constantly to assimilate calcareous matter without separating from the weak proportions of organic substances which fill it, so we cannot appreciate the phenomena of nutrition, which take place here, than by the changes of density which it presents with progress of age.

The chemical composition of the enamel resembles that of ivory, preponderating, however, in calcareous salts. We append the results of Bibra's analyses.

| | Molar of a woman aged 25. | Molar of an adult man. |
|---|---|---|
| Phosphate of lime, with traces of fluoride of calcium, | 81.63 | 89.82 |
| Carbonate of lime, | 8 88 | 4.37 |
| Phosphate of magnesia, | 2.55 | 1.34 |
| Soluble salts, | 0.97 | 0.88 |
| Organic substances, | 5.97 | 3.39 |
| Fat, | Traces, | 0.20 |
| | 100.00 | 100.00 |
| Organic substances, | 5.95 | 3.59 |
| Inorganic materials, | 94.16 | 96.51 |

### SECTION III.—*Cement.*

*Synonyms.* Osseous cortical, (Tenon,) *crusta petrosa,* (Blake,) cement, (Cuvier, Duvernoy, etc.,) osseous substance, (Erdl,) cortical substance, (Purkinje, Müller, Oudet,) *tooth bone,* (English.)

The cement, the least considerable part of the dental organ, is a true osseous substance. Its existence is nearly constant in the superior species of animals, but its volume is very variable. In the horse, elephant, dolphin and whale, its thickness is very great, and in the last two animals, it nearly attains to the volume of the dentine; in man, carnivorous animals, and rodents, it forms a thin covering about the root. In ruminants and pachyderms, it unites the different teeth in one mass, and in the human species sometimes embrace the roots of all of the molars.

In the human teeth the cement invests the whole surface of the roots. Commencing at the neck by a thin border it is prolonged over the enamel for a short distance, and spreads over the whole root, becoming thicker as it approaches the extremity, reaching the thickness of three to four millimetres. (pl. ii, fig. 4, *e.*) Its exterior aspect is very analogous to bone; it is yellowish and opaque, and in density is near to that of osseous tissue, and inferior to that of dentine. Closely attached to the exterior surface of the dentine, it exactly fills all the anfractuosities which it presents, so that the line of demarcation between these two tissues becomes almost inappreciable. Its external surface, covered by little nodosities, is covered by the alveolo-dental membrane, which here performs the office of a true periosteum, and from which vessels communicate with the cemental tissue.

The cement develops contemporaneously with the roots which it covers, and presents a continuous increase. Thus from being very delicate at the beginning of its formation, its volume increases with age and becomes considerable in old age, a circumstance which might serve to explain how certain teeth of old persons maintain themselves in their alveoli; thanks to the cement which surrounds them, and notwithstanding the complete disappearance of the central pulp.

Like bone, the cement is composed of fundamental substance and osseous cavities, (osseous corpuscles of Purkinje,

(*ostéoplaste* of Robin.) The canals of Haver, which are numerous in the cement of pachyderms and ruminants, are only met with in man, when the teeth acquire a certain thickness, at the ends of the roots for example, and principally in the hypertrophied masses, known under the name of exostosis which is so common.

The fundamental substance of the cement, (pl. ii, fig. 6, *a*, *a*,) is homogeneous or finely granular and diaphanous. In the neighborhood of the neck, (pl. ii, fig. 4, *d*,) where it is not osteoplastic it is thin, transparent and friable; we also often meet with it marked by striæ, fissures, etc. In the thicker parts it sometimes presents the appearance of stratified layers, common in the osseous tissue, and under these circumstances we may also observe the canals of Haver, the openings of which serve as common centres to the stratifications of the osseous substance.

The osseous corpuscles or osteoplasts are ordinarily arranged in the interior of the cement in a very irregular fashion, (pl. ii, fig. 6, *b*, *b*;) it does not then become necessary, with Kölliker, to discuss them in reference to their different form and divers characters, as we would with bone. Their number is always in proportion to the thickness of the layer of the cement. Their direction, according to Hannover, is such that their great diameter is presented perpendicularly to the axis of the tooth, whilst, according to Kölliker, the diameter is parallel to this axis. We have always seen them arranged without any order, without determined direction, and arranged here and there in the thickness of the cement. The osteoplasts only take a direction parallel to the contour of the stratified lamella, where we also meet with the Haversian canals at the same point; they there offer a more regular form and arrangement.

The ramifying canaliculi of the osteoplasts participate oftenest in the irregularity of the cavity, thus they may be seen to take the most strange directions in pl. ii, fig. 6, *c*. In certain cases they are all found on one side resembling a tuft of moss (Tomes ;) at other times they are all in one part of the cavity, and sometimes they are entirely wanting. In some places the canaliculi are seen in the directions of the exterior surface of the cement, and the alveolo-dental membrane from which they, without doubt, form the material of nutrition.

The dimensions of the osteoplasts are difficult to determine, yet their diameter seems at least to be from 0mm 03 to 0mm 06, in the largest. Kölliker* describes some of them as so elongated that one may compare them to the canaliculi of ivory, an analogy in which one cannot be mistaken, says he, and which establishes an insensible transition between cement and dentine. We have never observed this disposition and we believe in such a nice limit between these two substances that the parts of either never pass their respective boundaries, (pl. ii, fig. 6, *g*.) The same author also describes anfractuous spaces in the cement, which he considers as pathological productions without specifying their characteristics.*

The communication of the osteoplasts and their divisions with the terminal branches of the dental canaliculi, has been announced by several writers. Kölliker even indicates, as we have just seen, a special system of intermediary canals to the two others, and intended for the establishing of anastomoses. Hannover denies this, and founds his opinion on the fact that the cement and the ivory are separated by a peculiar matter, the *stratum intermedium*, a transformation of the *membrana intermedia*, which is opposed to this communication. Without invoking this last intervention, which we have always rejected, we do not think we can admit this union, which we have besides never observed, between the canals of the ivory and those of the cement; these two substances are very distinct, their structure and their chemical composition are very different, the two modes of nutrition and development of

* Histol. Hum., p. 424.

these two tissues are independent and no physiological condition, finally, can support this arrangement.

The chemical constitution of cement, nearly indicated by that of bone, is as follows, according to the analysis of Bibra:

| | IN MAN. | OXEN. |
|---|---|---|
| Organic substances, . . . . | 29.42 | 32.24 |
| Inorganic substances, . . . | 70.58 | 67.76 |
| | 100.00 | 100.00 |

These substances studied in the cow, have given:

| | |
|---|---|
| Phosphate of lime and fluoride of calcium, . | 58.73 |
| Carbonate of lime, . . . . . . . | 7.22 |
| Phosphate of magnesia, . . . . . | 0.99 |
| Soluble salts, . . . . . . | 0.82 |
| Cartilage, . . . . . . . . | 31.31 |
| Fat, . . . . . . . . . . | 0.93 |
| | 100.00 |

### CHAPTER II.—*Soft Parts of the Teeth.*

On the head of soft parts of the adult teeth, we comprehend the dental germ and the alveolar dental membrane.

### SECTION I.—*Dental Pulp.*

The dental pulp in the adult is nothing else than the dental papilla of the fœtus, greatly increased in size by the progress of development; it occupies the central cavity of the dentine, and is prolonged into the roots through the canals with which these are traversed. Exactly moulded to the walls of the cavity which contains it, it represents the tooth which covers it; thus in the interior of the canine it is spindle-shaped, and in the incisors it is shaped *en biseau*, and in the molars it presents a number of conical projections equal to the number of tubercles of the crown. Its volume gradually diminishes with age, and in old age is reduced to a thin, elongated fillet, occupying the middle of the central cavity of the tooth. Finally it entirely disappears towards the term of life, when the incessant production of the ivory elements has entirely filled up the cavity which it occupies.

The dental pulp is a soft, reddish or roseaceous organ, and does not offer an adherance to the dental parietes.

Examined chemically by Professor Wurtz.* it has been found to be impregnated with a strongly alkaline fluid, and containing, when in dissolution, a peculiar albuminous matter. This matter is the modification of the albumin which is formed by the action of the alkalies upon this principal. It precipitates by acetic acid, which distinguishes it from normal albumin. The liquid which holds it in solution, is incompletely coagulated by heat; it is also precipitated by mineral acids, tannin, metallic salts, such as subacetate of lead, sulphate of copper, sublimate; alcohol coagulates it in thick flakes. The alkalinity of this liquid excludes the idea of supposing the presence of phosphate of lime in a state of simple solution, it seems more probable that this salt is intimately combined with the albuminous matter itself.

Incinerated, the dental pulp has given, in the hands of the same chemist, a strong alkaline residue, in which only the *traces* of phosphate of lime were discovered.

In the histological point of view the dental germ of the adult differs only in a slight degree from the same organ in the fœtus. In short, it is found to be composed of a close fibrous web with homogeneous amorphous matter interposed; it is delicately granular and transparent, and within it embryoplastic nuclei in all the periods of their evolution may de observed. The nuclei are less voluminous however than in the fœtus, but their different characteristics are identical. We no longer find, in the organ, the calcareous masses and crystals of hematoidin which we have described as in the fœtus; yet we can still show the cells of dentine at the surface of the germ, arranged as we

* See l'Union Medicale, 25 Nov., 1856.

have indicated, and forming a continuous layer, which Kölliker regards as epithelial.

The vessels of the dental pulp are extremely numerous, partaking of the red color of this part. The pulp receives as many vascular and nervous filaments as the tooth has roots.

These vessels penetrate into the organ, and ramify, terminating at a certain distance from the surface *en anse.* The capillaries are, according to Kölliker, from 0mm 009, to 0mm 010 in size; they appertain to the third variety of M. Robin, and form the reticulations where they become veins. The dental pulp seems to be without lymphatic vessels, but they present an extremely rich system of nervous ramifications. In each root a nervous filament penetrates, according to Kölliker,* from 0mm to 002, to 0mm 003 in size. These fibres terminate in loops, (Wagner,) or by conical extremities, or swelled like a button.

### SECTION II.—*Alveolo-dental Membrane or Periosteum.*

The dental periosteum is a thin membrane interposed in the alveolus between the tooth and the jaw, and is intimately adherent to the cement, which it entirely covers. It is formed by the thick, external envelop of the follicle, which, as we have seen, is strongly attached to the neck of the tooth, where it commences, and where it often contracts an intimate attachment with the tissue of the gum, and of which it seems to be a continuation; this last circumstance, in the extraction of the teeth, sometimes causes tearing of the gum when care has not been taken to isolate the tooth from the soft parts. It then proceeds towards the end of the root, sending forth here and there, several fibrous bridles, which are attached to the alveolar walls, and it finally reaches the end of the root, where it encounters the vessels and nerves of the tooth, upon which it is prolonged, forming their sheath, without folding, as has

* See Histol. Hum., p. 426.

been believed, into the dental cavity and covering the surface of the pulp. These vessels are not accompanied by the periosteum, but come into immediate contact with the ivory.

The structure of this substance resembles that of the mucous membrane and osseous periosteum, and may be considered as something intermediate. It is composed of a simple fibrous web, without elastic elements, pervaded by a rich, vascular net-work, and by numerous nervous ramifications, formed, according to Kölliker, by large tubes. The vascularity of this membrane and its richness in nerves, explains the frequent inflammations to which it is subject, and the lively pain which accompanies them.

The alveolo-dental membrane is susceptible of presenting a great number of alterations which have yet been little studied, and in old age it seems to undergo a sort of gradual hypertrophy, which, perhaps, results, with other causes, from the physiological decadence of the dental organ.

### EXPLANATION OF THE PLATES.

Plates I and II, as here published, constitute Plate I in original work; and Plates III and IV, Plate II; bearing this in mind, the reader will have no difficulty in understanding the references.

### PLATE I.

FIG. 1.—Vertical section of the dental follicle of a provisory canine, from a fœtus of about six months, after the beginning of dentification.

*a.*—External envelop of the follicle.

*b.*—Internal envelop of the follicle.

*c.*—Pulp, or enamel germ, with its embryoplastic nuclei and their prolongations.

*d.*—Cells of the enamel calcified, and already transformed into prismatic columns.

*e.*—Cells of the enamel arranged in a continuous layer (adamantine membrane.) Some of these cells, in approaching the superior extremity, show the commencement of transformation; their nuclei atrophying, and the transverse striæ becoming visible.

*f.*—A small part of the exterior convex surface of ivory, from which the enamel detaches itself; the orifices of the canaliculi may be seen, and also some of the canaliculi stretched out from them.

*g.*—Section of the dentine subjacent to the preceding layer, pervaded by tubes, and representing a layer of dentine cells less completely developed than the last.

*h.*—Continuous row of dentine cells, the intervals between forming the canaliculi, which continue into the ivory.

*i.*—Dental germ with its embryoplastic elements within a granular amorphous matter.

*j.*—Open space filled by the liquid of the follicle.

*k.*—Vascular system of the ivory germ.

FIG. 2.—Thin section of the dental follicle before the beginning of dentification; the embryoplastic elements may be seen in their reciprocal arrangement; none of them as yet present prolongation. (Magnified 300 diameters.)

FIG. 3.—The same elements isolated, (300 diameters.)

FIG. 4.—The same elements, the commencement of fibro-plastic ramifications, (300 diameters.)

NOTE.—The centres of the nuclei in the three last figures, and also in the point *i*, in the 1st, are a little too strongly marked; the reader will please rectify this error in the drawing.

FIG. 5.—Vertical section of the ivory germ, and a thin cap of dentine which covers it, (300 diameters.)

*a.*—Summit of the cap of dentine seen from its convex surface. This lamella is entirely developed; one may catch glimpses of the canaliculi and the route they take.

*b.*—Section of dentine under the above, and less developed.

*c.*—Dentine cells with the origin of the canaliculi which create their intervals, and which extend into the preceding layer. (Glycerin has dissolved their nuclei.)

*c'.*—Cells subjacent to the above, and in process of development.

*d.*—Dental germ with the elements which compose it.

*e, e.*—Hematoidin crystals *en aiguilles,* in the interior of the organ, and indicating a great nutritive energy.

NOTE.—When in the microscopic examination of the dental germ, we approach its adherent base, and consequently, removing from the parts already covered by dentine, we find an increasing number of fibro-plastic, fusiform or ragged bodies which do not exist in that portion of the bulb represented in this figure ; but they may be seen in the interrupted extremity of the preparation which brings the internal adherent sack to the organ by degrees, and at last, in the points where this adherence is effected, the fibro-plastic bodies and the amorphous matter diminish in quantity, leaving room for the laminar fibres, which establish a direct continuity between the elements of the bulb and those of the membrane.

FIG. 6.—Lower face of the thin border of a cap of dentine in process of formation. (Magnified 500 diameters.)

*a.*—Dentine completely formed with its tubes, which may be seen between the intervals of the cells.

*b.*—Dentine cells seen from their base, and in process of calcification: we observe that each of them is surrounded by whitish, transparent furrows, produced by the gluing together of new cells, form the complete canals constituting the lateral branches of the canaliculi. We also meet with rounded orifices of the tubes at the angles of some of the cells.

*c.*—The same cells less developed, and still very granular.

FIG. 7.—Isolated cells of the dentine with all the forms which they present. A large number of them are provided with ordinary simplo-prolongations, which are rarely bifed; some others are more or less regular, and with out prolongations. All of them contain a round or oval nucleus, which going out of the cell, tend to become free. (400 diameters.)

### PLATE II.

FIG. 1.—Germ of the enamel, composed of its pulp or gelatinous part of its enamel cells. (500 diameters.)

*a.*—Finely granular and transparent, amorphous matter mixed with fibro-plastic bodies.

*b.*—Stelliferous fibro-plastic bodies.

*c.*—Prolongations of the same fibro-plastic bodies.

*d.*—Amorphous matter becomes more granular in the neighborhood of the cells.

*e.*—Cells of the enamel arranged in continuous layers, (adamantine membrane.)

FIG. 2.—Enamel cells isolated. (500 diameters.)

FIG. 3.—Enamel cells in process of calcification; the nucleus has entirely disappeared, as also the granulations.

FIG. 4.—Vertical section of a permanent incisor. (10 diameters.)

*a.*—Enamel with its *contour lines,* indicating its formation in superimposed layers.

*b.*—Ivory with its lines of contour, indicating the same disposition.

*c.*—Central cavity of the tooth.

*d.*—Neck of the tooth; we may here see the cement pushed up under the border of the enamel.

*e.*—Cement.

FIG. 5.—Vertical section of an adult incisor intersecting a part of the enamel and a part of the ivory. (350 diameters.)

*a.*—Prismatic columns of the enamel arranged in superimposed layers.

*b.*—Contact lines of the layers.

*c.*—Anastomotic cavities which unite the terminal extremities of the canaliculi.

*d.*—Interglobular spaces seen in profile, and in which we see a certain number of canaliculi.

*e.*—Dental canaliculi.

*f.* Line of demarcation between the ivory and the enamel.

FIG. 6.—Vertical section of an adult tooth intersecting the cement and the ivory, (350 diameters.)

*a, a.*—Fundamental substance of the cement on the osseous parts, marked by granular striæ, indicating the commencement of stratification.

*b, b.*—Osseous corpuscles or osteoplasts, irregularly arranged within the fundamental substance, and presenting the most varied forms.

*c.*—Ragged canaliculi of osteoplasts.

*d.*—Continuous granular layer, subjacent to the cement and enamel, (see fig. 5, *c*,) and arranged within the ivory; they are composed of cavities which form that which we call the *anastomotic spring* of the dental canaliculi.

*e.*—Anastomosis of two dental canaliculi *en anse.*

*f.*—Dental canaliculi with their ramifications and their anastomoses.

*g.*—Shaded limit, separating the ivory of the cement, and opposing any communication between these two substances.

FIG. 7.—Lower face of a cap of dentine in process of formation, covered by dentine globules of varied and progressively growing dimensions. (300 diameters.)

*a.*—Large globules of dentine traversed by canaliculi in the direction of the surface of the preparation.

*b.*—Orifices of the canaliculi with a part of their length lost in lower part.

*c.*—Little globules recently formed, which will acquire the size of the others by degrees.

Fig. 1.

Fig. 2.

Fig. 3.

Fig. 4.

T. Sinclair's lith, Philad[a].

Fig. 6.

Fig. 7.

Fig. 5.

T. Sinclair's lith, Philad[a].

Fig. 1.

Fig. 2.

Fig. 3.

Fig. 4.

T. Sinclair's lith, Philad[a].

Fig. 5.

Fig. 6.

Fig. 7.

T. Sinclair's lith. Philad^a

## Figures and Tables

**Fig. 1. f1:**
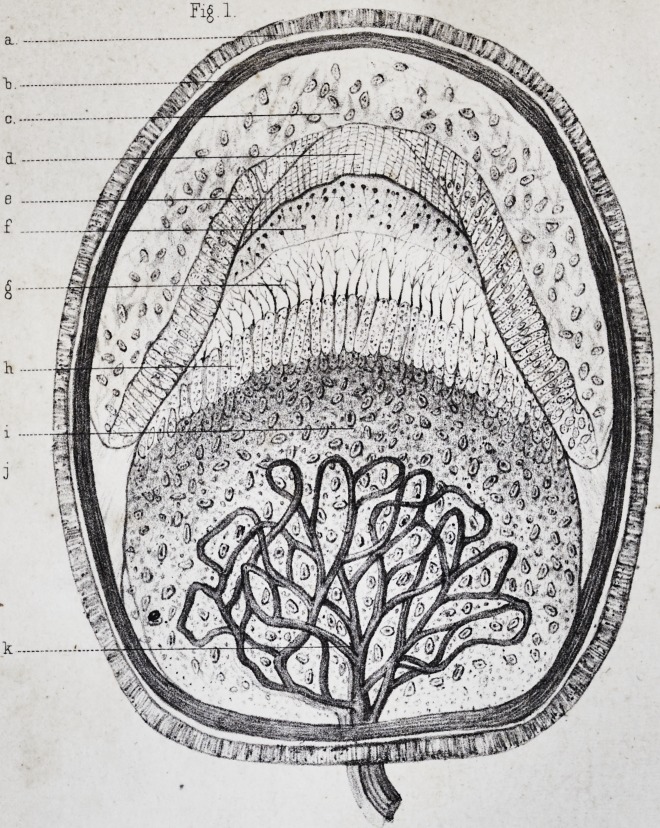


**Fig. 2. f2:**
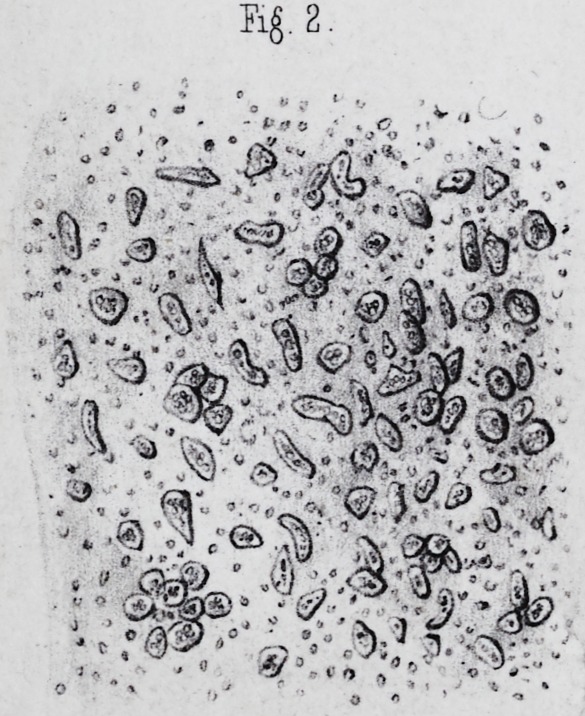


**Fig. 3. f3:**
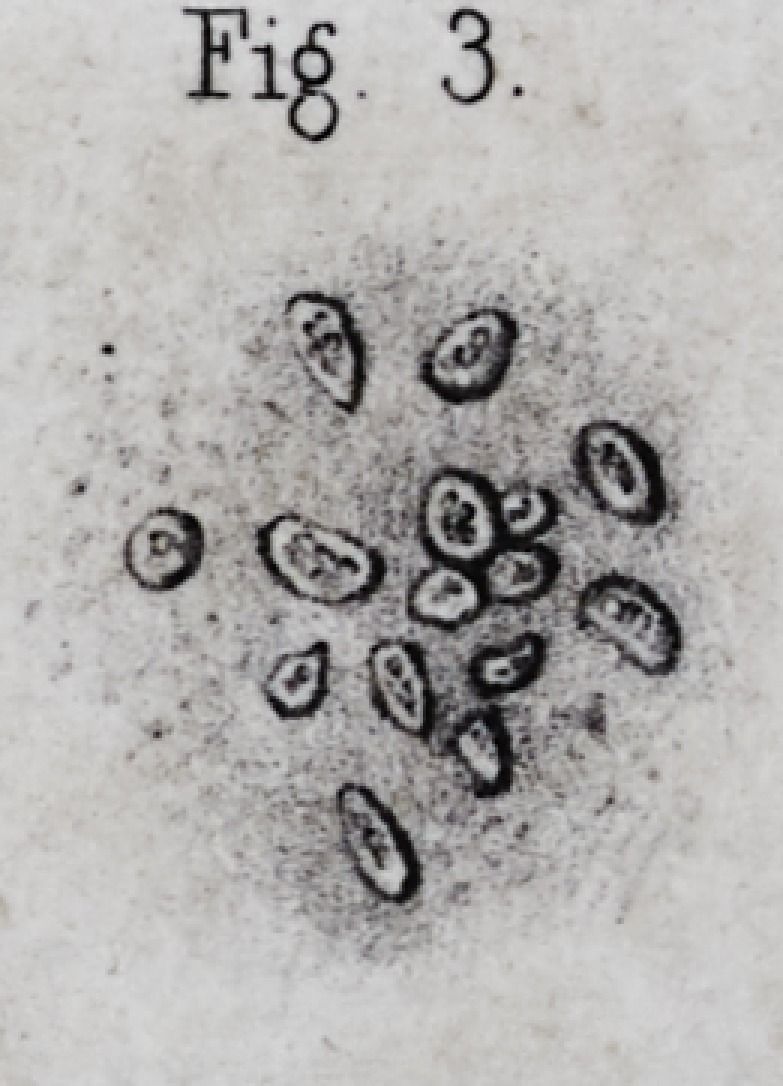


**Fig. 4. f4:**
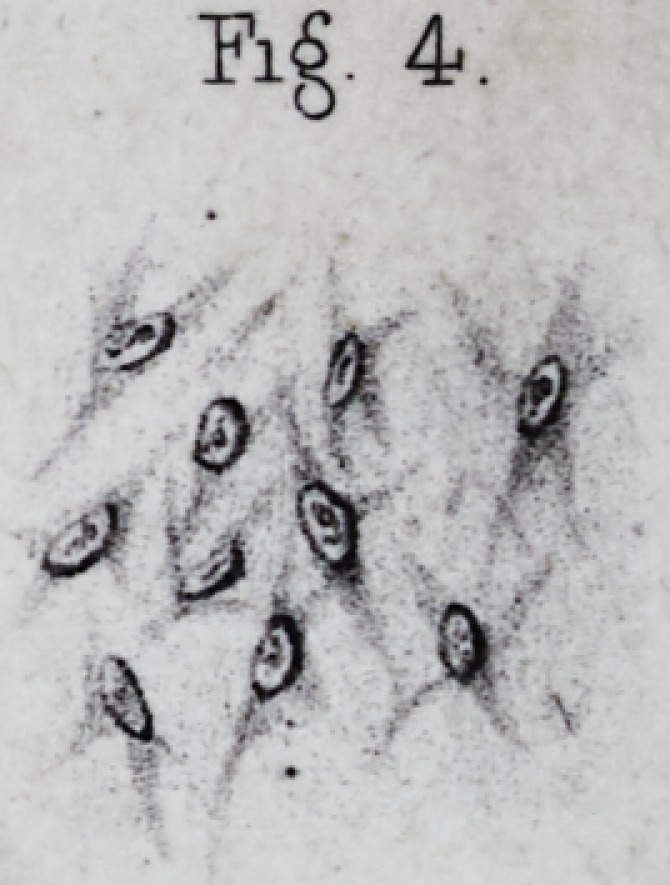


**Figure f5:**
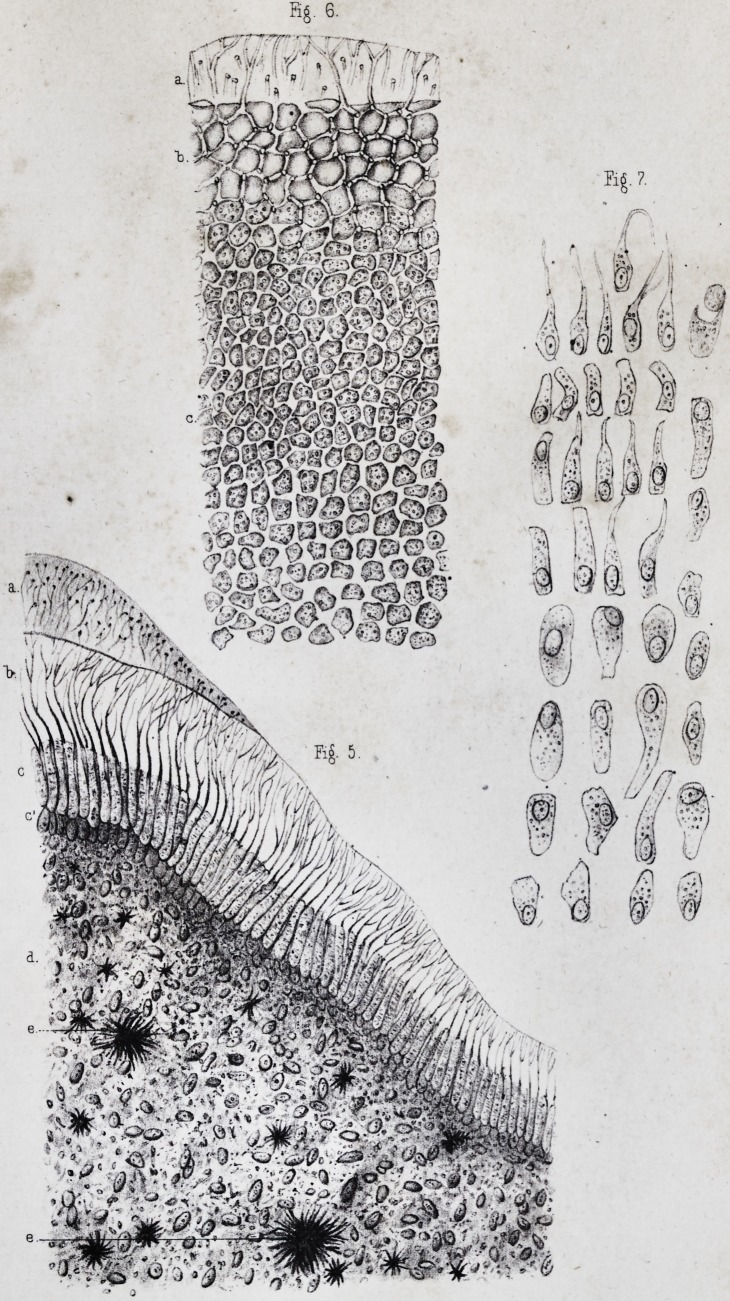


**Figure f6:**
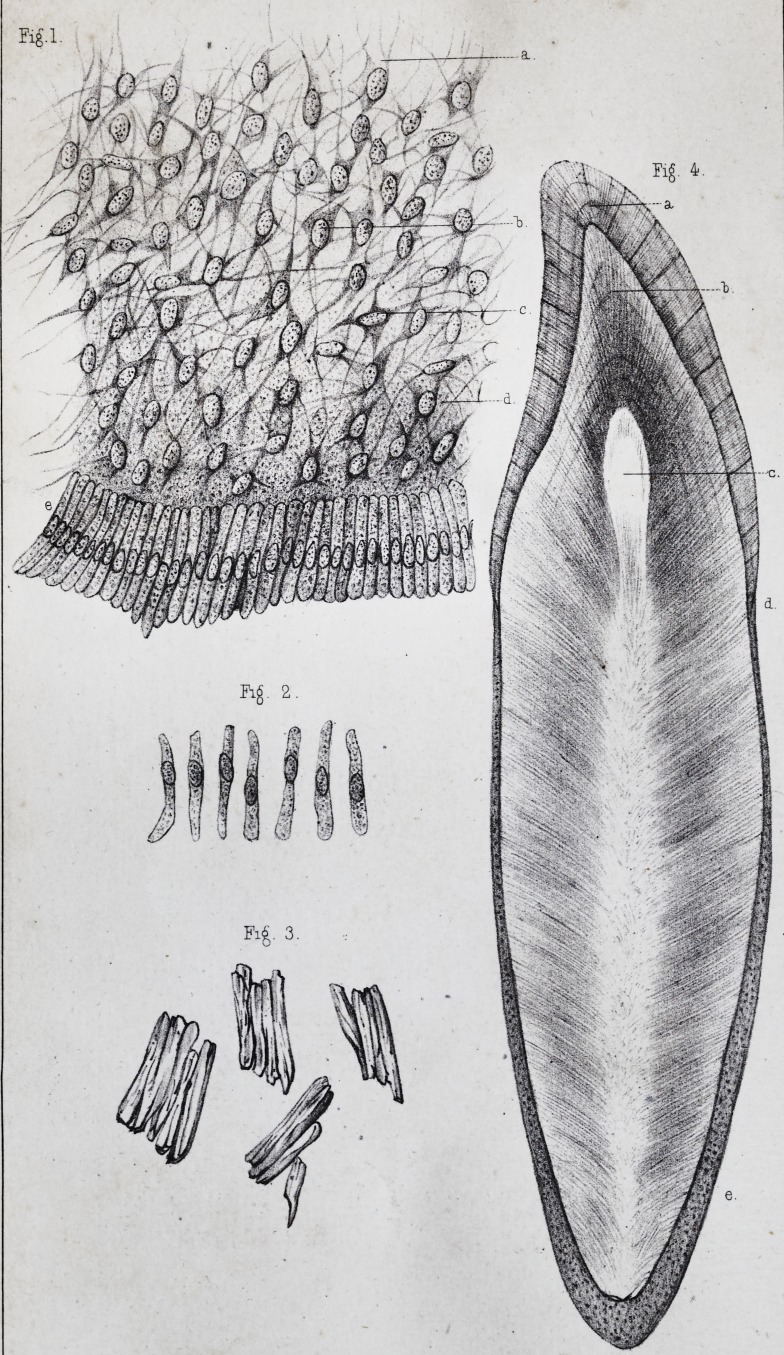


**Fig. 5. f7:**
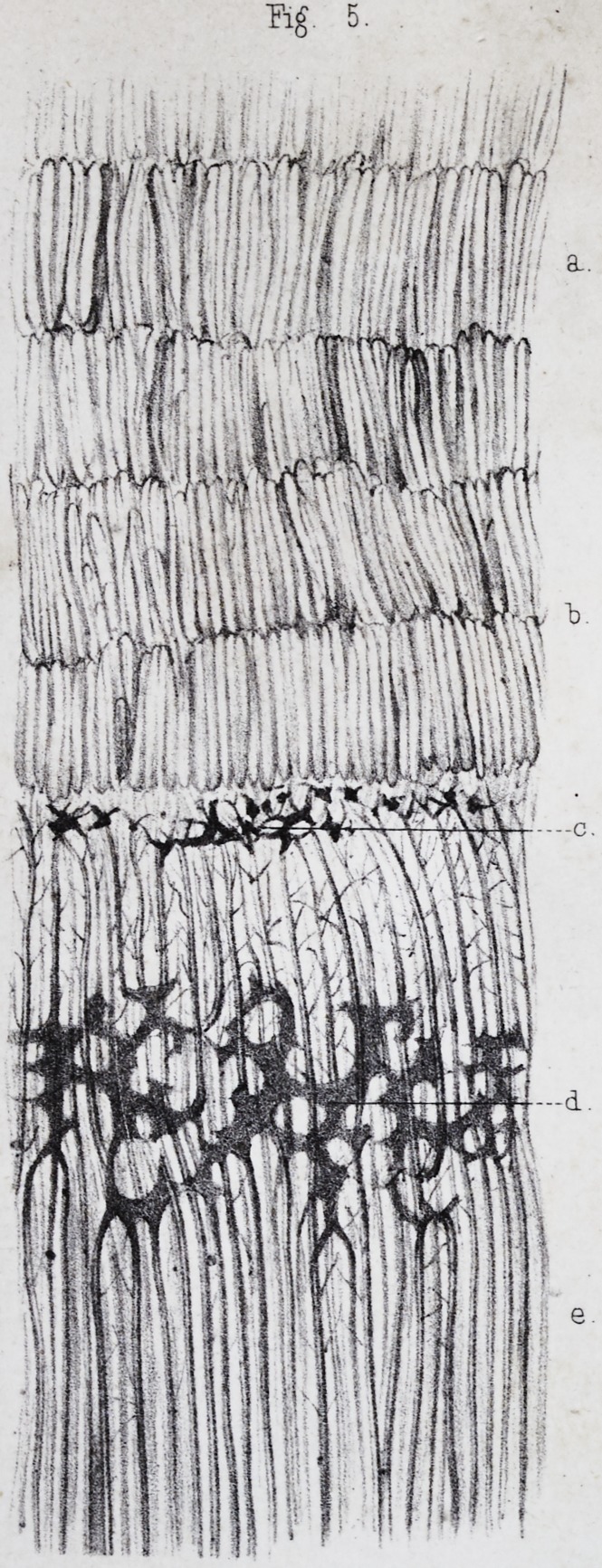


**Fig. 6. f8:**
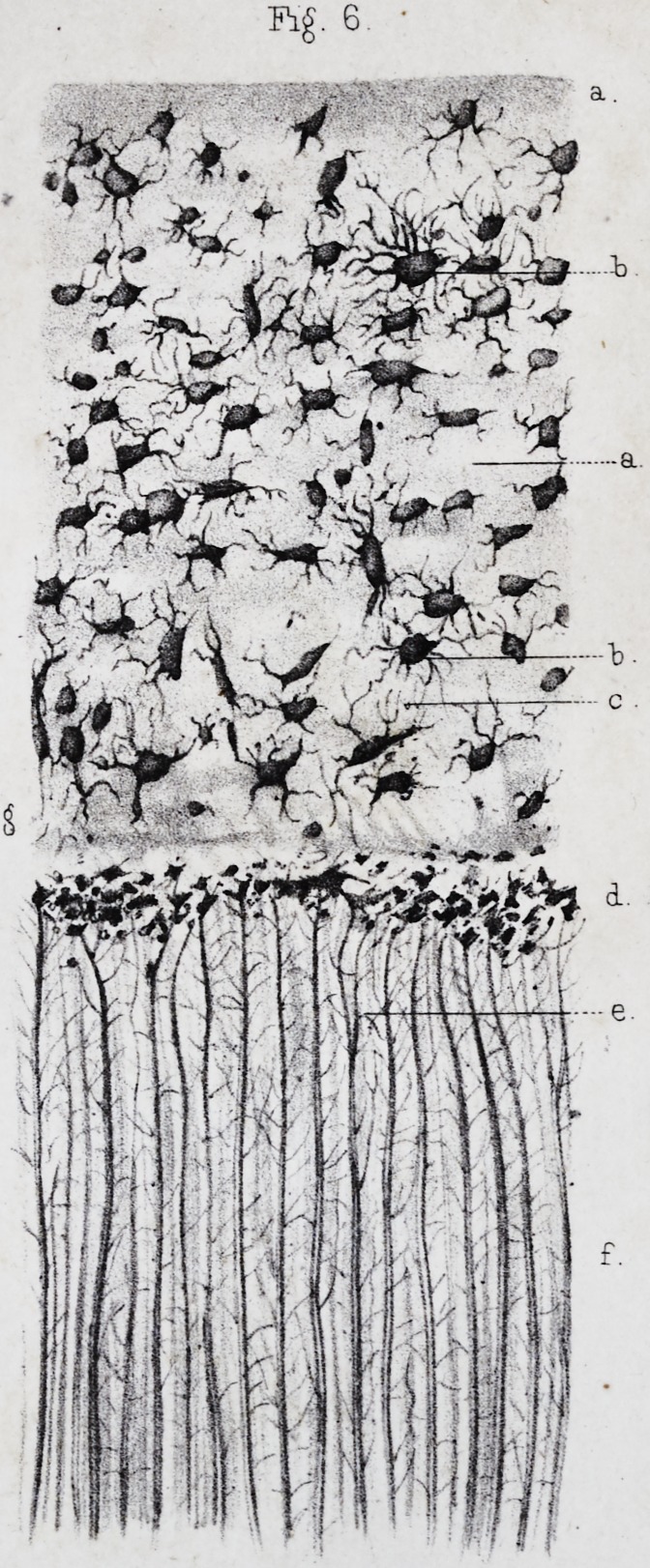


**Fig. 7. f9:**